# Calibrating TDDFT Calculations of the X-ray Emission Spectrum of Liquid Water: The Effects of Hartree–Fock Exchange

**DOI:** 10.1021/acs.jctc.3c00728

**Published:** 2023-10-03

**Authors:** Thomas Fransson, Lars G. M. Pettersson

**Affiliations:** Department of Physics, AlbaNova University Center, Stockholm University, 109 61 Stockholm, Sweden

## Abstract

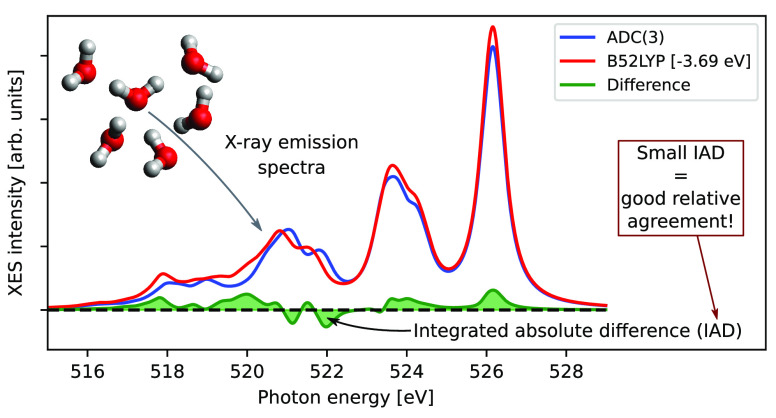

The structure and dynamics of liquid water continue to be debated, with insight provided by, among others, X-ray emission spectroscopy (XES), which shows a split in the high-energy 1b_1_ feature. This split is yet to be reproduced by theory, and it remains unclear if these difficulties are related to inaccuracies in dynamics simulations, spectrum calculations, or both. We investigate the performance of different methods for calculating XES of liquid water, focusing on the ability of time-dependent density functional theory (TDDFT) to reproduce reference spectra obtained by high-level coupled cluster and algebraic-diagrammatic construction scheme calculations. A metric for evaluating the agreement between theoretical spectra termed the integrated absolute difference (IAD), which considers the integral of shifted difference spectra, is introduced and used to investigate the performance of different exchange-correlation functionals. We find that computed spectra of symmetric and asymmetric model water structures are strongly and differently influenced by the amount of Hartree–Fock exchange, with best agreement to reference spectra for ∼40–50%. Lower percentages tend to yield high density of contributing states, resulting in too broad features. The method introduced here is useful also for other spectrum calculations, in particular where the performance for ensembles of structures are evaluated.

## Introduction

Of all compounds necessary for the existence of life on Earth, water is the most important and most common. As a result, the nature of its structure and dynamics has been studied in great detail, and a number of unusual behaviors have been identified.^1^ These include properties such as the density increase upon melting, high surface tension, decrease in viscosity under pressure below 46 °C, and many more.^1−3^ These anomalous behaviors become enhanced in the supercooled region, but they are also present under ambient conditions. A consensus on the origin of these properties has not yet been reached, but it is expected to be found in the minute details of the structure and dynamics of the hydrogen-bonding network.^4,5^ The structure of liquid water was long suggested to be fluctuating around an on average tetrahedral motif, but there is mounting experimental^6−9^ and simulation^10−12^ evidence that water can exist in two distinct liquid forms with a liquid–liquid phase transition in the deeply supercooled and pressurized region of the phase diagram. The present debate contrasts the tetrahedral model with a continuum of distorted hydrogen bonds against a two-state model with two distinct components. A two-state picture of water leading to predominance at ambient conditions of close-packing of molecules (HDL, favored by entropy), but with local fluctuations into tetrahedral environments (LDL, favored by enthalpy), is fully consistent with thermodynamics, where such models have been shown to quantitatively reproduce the thermodynamic properties of water.^13,14^

For the investigation of the electronic and atomic structure of molecular materials, X-ray spectroscopies provide a number of highly element-specific probes, capable of addressing occupied states, unoccupied states, and more.^15−17^ Included here is X-ray emission spectroscopy (XES), for which the fluorescence decay of core-ionized or core-excited molecules provides information about occupied states. Focusing on the use of core-ionized intermediates (i.e., nonresonant XES), X-ray emission occurs when a valence electron refills the initial core-hole, and thus grants insight into the valence states. Due to the large differences of core-orbital energies of different elements, these techniques are highly element-specific and, combined with chemical shifts due to the chemical environment, can thus be utilized to investigate the local electronic structure at specific atoms in a sample.^18^ Nonresonant XES in the soft X-ray region, i.e. up to about 2000 eV, has been extensively used for the study of low-Z elements and compounds, such as the surface chemical bond,^19^ the structure of liquid water,^17^ CO oxidation at metal surfaces,^20^ and searching for fingerprints of amino acids.^21^ Despite the short lifetime of core-ionized states (fs), it has been noted that core-hole-induced dynamics influence XES spectra, particularly for proton dynamics.^21−28^ The spectroscopy has thus enabled insights into structure and dynamics of the hydrogen-bonding network in, among others, liquid water,^17,24,26,29−34^ methanol,^22^ and ethanol,^23^ as well as in the investigation of the properties of many other liquids.^35,36^ However, in order to achieve a fundamental understanding of the systems and properties under investigation, quantum chemical calculations are vital, and the concomitant development of reliable theoretical tools is thus a necessity.^18,37^

The connection between the structure and dynamics of water and the X-ray emission spectra has been extensively investigated, but no unambiguous interpretation has yet been found.^17,26,27,29,31,32,38,39^ For nonresonant excitation, the XES spectrum of gas phase water exhibits three main features that arise due to electron transitions from the valence bonding 1b_2_, 3a_1_ and nonbonding 1b_1_ molecular orbitals, where the 1b_1_ peak is sharp, while the 1b_2_ and 3a_1_ peaks are smeared due to more significant vibrational contributions. In contrast to the gas phase, the XES spectrum of liquid water shows a split of the lone-pair 1b_1_ peak, usually labeled as 1b_1_′ (low energy) and 1b_1_″ (high energy). The source of this peak split remains to be fully resolved, with the two main current interpretations proposing that the split originates from proton dissociation in the core-ionized state, or as a result of liquid water consisting of two local structural motifs.^17^ Using theory, the influence of core-hole dynamics on the XES spectrum has been studied using, e.g., semiclassical Kramers–Heisenberg,^22,23,40,41^ and detailed wave packet dynamics on, e.g., gas phase water.^42^ Extensive experimental measurements have been performed in which the influence of temperature variation, isotope substitution, and variations in chemical surrounding have been investigated.^17^ These studies demonstrate that the intensity ratio between the two features in the double structure is sensitive to hydrogen bonding, with a relative intensity of 1b_1_′ increasing with hydrogen-bond coordination.

Modeling of X-ray emission spectra has been performed using density functional theory (DFT),^23,25,32,41,43−46^ time-dependent DFT (TDDFT),^38,45−47^ ground state DFT,^48,49^ multireference methods,^50^ Green’s function theory,^51^ coupled cluster (CC) theory,^47,52,53^ the algebraic diagrammatic construction (ADC) scheme for the polarization propagator,^54,55^ and more.^18^ A protocol which has been successfully applied is to construct a core-ionized reference state, and use linear response theory to calculate transitions into the core-hole.^52^ This gives access to transition energies, intensities, and excited state properties for all transitions to the core-hole in a single calculation, thus avoiding any need of separate calculations for each transition (albeit one for each possible core-hole is still needed, and we note that they in most cases should be localized^56^). In order to apply such an approach, the construction of a core-ionized reference state is necessary, and multiple schemes for avoiding its collapse to a valence-ionized state have been developed, such as the maximum overlap approach.^57−59^ The calculation of XES from core-ionized reference states has been considered using time-dependent DFT (TDDFT),^38,45−47,60,61^ equation-of-motion coupled cluster singles and doubles (EOM-CCSD),^47,52,53^ and ADC.^54,55^ It has been noted that the relaxation (and thus the performance of different methods) is markedly different compared to that of core-excitation processes, or for valence transitions.^54^ Among these methods, TDDFT exhibits a low computational cost, but the issue of self-interaction plagues calculations of both X-ray absorption and emission spectra.^18,45−47,62,63^ This is due to spurious self-interactions in the final state and typically leads to X-ray emission features being too high in energy. Nevertheless, using scalar shifts of spectral features or exchange-correlation functionals tailored for X-ray properties, TDDFT continues to be utilized for the simulation of XES spectra of both low-Z^21,52^ and transition metal elements.^64−66^ We note that approaches for removing or minimizing the self-interaction error are being developed, such as using electron-affinity TDDFT^67^ or correction schemes based on many-body perturbation theory.^68^

Here, we investigate the performance of different methods for calculating X-ray emission spectra of water. We have earlier shown that, already without including core-hole-induced dynamics, the computed 1b_1_ peak position of molecules in a well-defined tetrahedral environment (LDL-like) appears at lower energy than for molecules in highly disordered environment (HDL-like) and that the magnitude of the split is sensitive to the degree of tetrahedrality and disorder.^69^ The split is thus mainly determined by the initial H-bonding situation, if defined according to these criteria, and further enhanced (∼0.1–0.2 eV) by core-hole-induced dynamics.^28^ However, currently available simulation models of liquid water seem to generate a distribution of structures that, in terms of electronic structure, is intermediate between locally LDL- and HDL-like.^69^ Thus, an effort was made to perform high-level QM/MM calculations of XES, combined with computed X-ray absorption spectra (XAS) of the same structures extracted from MD simulations and, by simultaneously fitting to experimental XES, XAS and the measured O–O pair-distribution function, deduce a distribution of local H-bonding structures that would be consistent with experiment.^70^ However, in the XES calculations only 3 QM molecules could be included due to the cost of the RASSCF/RASSI wave functions. It is thus desirable to extend these calculations to larger QM models by using time-dependent density functional theory (TDDFT). Here, however, preliminary calculations have revealed a significant difference in spectral sensitivity between LDL- and HDL-like local structures to the amount of exact exchange, which is an issue that needs to be addressed before attempting the desired large-scale TDDFT calculations.^71^

Thus, we focus on the ability of TDDFT to reproduce reference spectra obtained by high-quality calculations using equation-of-motion coupled cluster singles and doubles theory and the ADC-scheme for the polarization propagator. We evaluate different exchange-correlation functionals for their ability to reproduce *ab initio* reference spectra both for highly structured (tetrahedral) and disordered (asymmetric) six-molecule water clusters. A measure termed the integrated absolute difference (IAD) is proposed,^72−75^ which probes the difference in broadened spectra obtained by different methods. The study is outlined as follows: we first discuss the different methods of calculating X-ray emission spectra, as well as introduce the IAD. This is followed by a presentation of the origin of the structures used and then by computational details on the spectrum calculations. Moving to results and discussion, we investigate the performance of the reference methods, discuss the properties of IAD, and test the performance of TDDFT with different standard functionals and with functionals with variable fractions of Hartree–Fock exchange. Additional details and complementary results can be found in the Supporting Information (SI).

## Methodology

The X-ray emission spectra are calculated as eigenstates of a core-hole reference state,^52−54^ which in turn is constructed by use of the maximum overlap method (MOM).^57−59^ For TDDFT, the Tamm–Dancoff approximation (TDA)^76^ is used, and a number of different exchange-correlation (xc) functionals have been applied, considering global and range-separated hybrids. An alternative approach for calculating X-ray emission spectra using only ground-state Kohn–Sham DFT is available (here called GS-DFT), where transition energies are calculated from molecular orbital (MO) energy differences and intensities from the transition dipole moment between MOs of the valence and relevant core state. This approach has been used with success for isolated molecules,^45,46,61,77^ but we here see that it gives too broad features for water clusters—in particular for fully H-bonded structures (see the SI). Among correlated *ab initio* methods for excited states, we here apply the algebraic diagrammatic construction (ADC) scheme for the polarization propagator and coupled cluster (CC) theory. In ADC, the polarization propagator is expanded in a perturbation series, where the poles and residues of its spectral representation correspond to excitation energies and transition amplitudes.^78−80^ The hierarchy of ADC methods is obtained by truncating the perturbation expansion at a desired order, with efficient implementations available for ADC(1), ADC(2), ADC(2)-x, and ADC(3), and recent development enabling the calculation of ADC(4) excitation energies.^81^ We note that the ADC(3) implementation used here is correct to third order in transition energies, but to second order in property gradients; as such, it is sometimes referred to as ADC(3/2). An alternative hierarchy of post-HF methods is available through the coupled cluster (CC) approach, which can be considered in either an equation-of-motion (EOM) or a linear response (LR) formalism. For X-ray emission spectrum calculations, ADC(2) and EOM-CCSD have been shown to yield good agreement with the experiment in terms of absolute energies, while ADC(2)-x yields good relative features.^52−55^ ADC(3) has been seen to yield less accurate relative and absolute features, but it has been noted that for calculations of gas phase water, considering 100 structures from *ab initio* MD dynamics, the resulting absolute features for ADC(2), ADC(2)-x, and ADC(3) are almost identical.^54^ For post-HF methods, issues related to the presence of unoccupied MOs with significant negative energy for the core-hole reference state can lead to instabilities, which become strong for small denominators (in absolute energy) in the MP2 energy correction.^55^ The smallest denominators have thus been tracked as a control to avoid such instabilities.

As a measure of the agreement between the spectra of two different methods, we apply the integrated absolute difference (IAD) as a statistic for comparing the calculated spectra. This measure was introduced to analyze experimental X-ray emission spectra of transition metal compounds and alloys to track spin and oxidation states.^72−75^ In these studies, the IAD is typically calculated with reference to some known material, and we adapt this here to provide a convenient measure of the correspondence between theoretical spectra. To calculate the integrated absolute difference of a broadened spectrum σ(ω) with respect to a reference spectrum σ_ref_(ω), the spectrum is first shifted in energy to align some predefined feature in the two spectra, yielding σ_shifted_(ω). The IAD is then given as
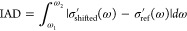
where σ_shifted_^′^(ω) and σ_ref_^′^(ω) are area-normalized to 1 over the energy interval ω_1_ to ω_2_. This thus produces a number ranging from 0 to 2, with 0 representing perfect agreement in (relative) spectrum features. The measure is constructed such that it probes the relative features in terms of both energy positions and intensities with a shift in energy and area-normalization ensuring that absolute energies and intensities do not contribute. While agreement in both absolute and relative features is clearly preferred, it has been seen that good absolute features do not necessarily correspond to good relative features.^82^ Note that the numerical value of the IAD will depend on the broadening scheme, energy shift, and energy interval, as will be discussed in more detail. However, provided a consistent choice of such parameters, the measure provides a convenient way of comparing the relative performance of theoretical methods, in particular, when considering ensembles of structures. Other spectrum descriptors are being developed, as relevant for, e.g. the use of machine learning for associating spectra with molecular structure.^83^

Water clusters were taken from ref (69), where structures were characterized in terms of the H-bond symmetry/asymmetry and the resulting 1*b*_1_ peak position. The present structures have been selected as highly tetrahedral (representing low-density LDL, local environment) and asymmetric (representing high-density HDL, local environment), featuring relatively short and well-defined or longer and asymmetric H-bonds, respectively, in order to investigate the spectra of these more extreme configurations. Tests on intermediate structures show that the results in terms of the IAD discussed here are valid for such species, as well. Additional calculations on gas phase water used the equilibrium structure of a single water molecule. Coordinates of all computed structures are available in the SI.

## Computational Details

The geometry of gas phase water was optimized at the frozen-core MP2 level of theory, using cc-pVTZ basis sets,^84^ as implemented in Q-Chem 5.2.^85^ Spectrum calculations of water clusters were run using a 6-311G** basis set,^86^ with the six core basis functions uncontracted,^47,54^ while the other oxygens were given an effective core potential (ECP) of the Stuttgart–Cologne type (a detailed description of how this was done technically can be found in the SI), and a 6-311G basis set.^87^ The two hydrogens bonded to the central oxygen were given a 6-311++G** basis set, and for the other hydrogens, we used 6-311G. Spectra of the isolated molecule were calculated using a cc-pVTZ basis set, including core-polarizing functions for the oxygen. For the water cluster calculations, a total of six water molecules were included in each cluster (selected larger clusters are shown in the SI), with spectra only calculated for the central molecule. As mentioned in the Introduction, the aim of the present study is to calibrate and determine prerequisites for reliably applying TDDFT to larger and more realistic clusters. The present cluster size was thus selected to allow for higher-level ADC and CCSD reference calculations. We further note that since nonresonant XES is charge-neutral, i.e. a core-hole is replaced by a valence-hole, it is less sensitive to cluster size than is XPS, which measures the difference between the neutral and core-ionized system and thus requires convergence of the response to the generated charge.

Convolution of the obtained intensities using a Lorentzian broadening function was performed to facilitate the analysis, using a half-width at half-maximum (HWHM) of 0.3 eV, unless stated otherwise. The coupled cluster calculations were carried out using Q-Chem 5.2,^85^ while the ADC results were obtained using the adcc software package,^88^ using SCF results obtained from pyscf.^89,90^ The smallest absolute values of the MP2 energy denominator for the core-hole reference states were noted to be above 0.1 au, and numerical instabilities due to near-singularities are thus not present in these calculations.^55^ TDDFT calculations were performed with PBE0,^91^ tailored versions of B3LYP^92^ and BHandHLYP,^93^ CAM-B3LYP,^94^ and from the QTP^95,96^ and SRC^97^ families of xc-functionals.

## Results and Discussion

### Performance of Reference Methods

**Figure 1:**
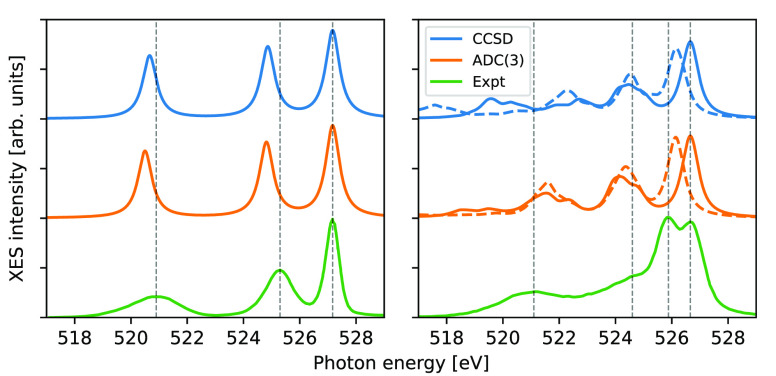
Left: X-ray emission spectra of gas phase water, compared to the experiment.^27^ Theoretical results have been shifted in energy by 0.00 and 0.87 eV for CCSD and ADC(3), respectively. Right: X-ray emission spectra of 10 HDL (solid line) and 10 LDL (dashed line) water hexamers, compared to experiment of liquid water.^34^ Theoretical results have been shifted by −0.36 and 0.49 eV for the CCSD and ADC(3), respectively. Dashed vertical lines indicate the approximate positions of the experimental features.

In order to investigate the performance of the reference methods, the ADC(3) and CCSD spectra of gas phase water and of 20 hexamers are shown in Figure 1, compared to the experiment.^27,34^ For the hexamers, 10 disordered (mimicking local HDL environments, henceforth labeled HDL) and 10 tetrahedral (mimicking local LDL environments, labeled LDL) clusters were considered, and the summed spectra of each selection is shown. This has not been done to reproduce the experiment, as the selected structures are few and small, we only consider static structures, and the clusters have been selected from trajectories in such a manner that clearly distinct tetrahedral and asymmetric structures are obtained. This is instead done to investigate the performance of different theoretical models for distinctly different structures. As such, the comparison here to experiment is to be taken as illustrative and not as any definitive demonstration of the ability of reproducing experiment on the liquid. Furthermore, the theoretical results have been shifted to overlap with the 1b_1_ feature for gas phase water, and with the high-energy 1b_1_ (1b_1_″) for the liquid. These shifts account for effects such as missing relaxation, basis set incompleteness, environment effects, and the lack of relativistic effects (which would here shift the absolute energies by ∼0.3–0.4 eV).^54,98^

The u6-311G** basis set has been seen to yield very similar results to when using cc-pVTZ with core-polarizing functions on the probed atom,^55^ as has been verified by separate calculations (results not shown). Basis sets can be formulated for core-ionized and core-excited states, using methods such as including functions from the next element,^99^ amending basis sets with core-polarizing functions,^100−102^ uncontracting core functions,^47,54,103^ using basis sets developed for NMR calculations,^104^ and forming new basis sets based on, e.g., Slater’s rules.^105^ Uncontracting core functions has been seen to be a relatively straightforward and cheap way of obtaining good accuracy,^55,103^ and all remaining results have been obtained using this scheme.

For the gas phase, the relative position and integrated intensities of the features are close to experiment, which has the 1b_2_, 3a_1_, and 1b_1_ features situated at approximately 521, 525.4, and 527.1 eV, respectively.^27^ The present calculations lack any dynamical effects, which influence the broadening of the peaks at different levels; vacancies in the bonding 1b_2_ and 3a_1_ orbitals lead to extensive vibrational broadening, whereas a vacancy in the nonbonding 1b_1_ orbital results in a sharper feature.

For the water clusters, the ADC(3) and CCSD hexamers yield 1b_1_ splits of ∼0.5 eV, which can be compared to the ∼0.8 eV split in experimental measurements of ambient liquid water.^34^ In general, calculated 1b_1_ splits are often too small, and the precise mechanism of this inconsistency is debated.^17,26−29,31−34^ Note again that the present calculations do not take into account effects of core-hole-induced dynamics, which would broaden the bonding 3a_1_ and 1b_2_ features. With this, we note that ADC(3) shows four distinct features, approximately corresponding to gas phases 1b_2_, 3a_1_, and the split 1b_1_. This is similar to the experiment, where 1b_2_ and 1b_1_ are distinctly visible, whereas 3a_1_ is more of a shoulder feature. By comparison, CCSD yields a more smeared out 1b_2_ peak, with features on both sides of the experimental 1b_2_ position. For ADC, it has been demonstrated that ADC(2) and ADC(2)-x yield superior X-ray emission spectra for isolated molecules, as compared to ADC(3),^54,55^ although a reported calculation on 100 water structures results in almost identical relative features.^54^ However, moving to the liquid phase, ADC(2) and ADC(2)-x show issues relating to poorer performance for doubly excited states, which here yield satellite states in the region of direct transitions. This results in corrupt spectra with features which are unphysically smeared out (see SI), and these methods are thus not suitable for this study. For ADC(3) and CCSD, the satellite states are shifted in energy and thus do not yield these complications.

### Definition of IAD

**Figure 2:**
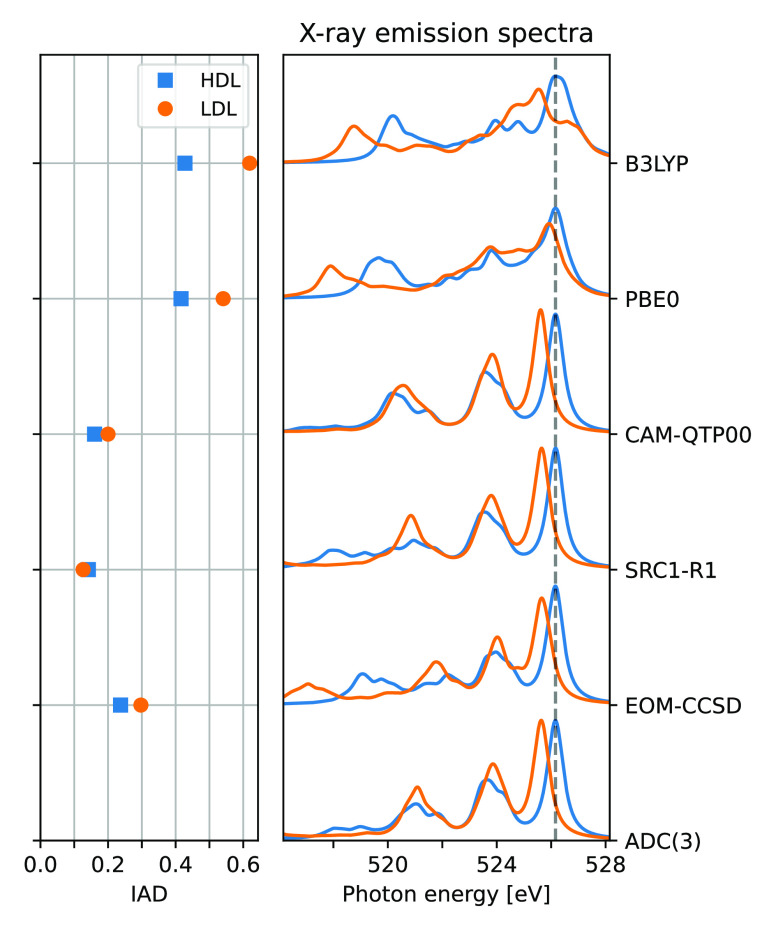
X-ray emission spectra and integrated absolute differences (IADs) of EOM-CCSD and TDDFT with four different xc-functionals, compared to the ADC(3) reference spectra. Spectra have been shifted to overlap with the 1b_1_ peak of HDL (horizontal dashed line), and the IADs have been calculated over the illustrated energy region. Applied energy shifts amount to, from top to bottom: −8.80, −6.51, −3.38, −4.08, and −0.85 eV.

The summed spectra of 10 HDL and 10 LDL structures, respectively, are shown in Figure 2, as obtained using EOM-CCSD and TDDFT with different functionals and compared to ADC(3) results; we remind the reader that the nomenclature HDL and LDL here only refers to the local coordination around the central molecule. The B3LYP and PBE0 functionals have been selected as representations of global hybrids, and the CAM-QTP00 and SRC1-R1 functionals have been included as examples of range-separated hybrids tailored for core transitions. Broadened spectra have been shifted to overlap with 1b_1_ of the HDL structures, and the IAD has been calculated over the energy range in the figure; the LDL spectra were shifted by the same amount. The 1b_1_ peak-split ranges from 0.24 to 0.53 eV, with the smallest value obtained using the B3LYP xc-functional. Looking at the IAD, values range from ∼0.1 up to ∼0.6, and they follow visible trends as well as discrepancies in 1b_1_ splits. IAD values for LDL structures are larger than for HDL structures for all calculations except SRC-R1, which partially reflects the use of an energy shift to align the 1b_1_ feature of the HDL cluster. Spectra for each individual structure are provided in the SI, as obtained using ADC(3), as well as the impact of the number of structures on the IADs and 1b_1_ splits. The IAD is seen to be more stable than the 1b_1_ split with respect to the number of structures as it considers integrated differences and not merely differences in peak positions. Furthermore, the SI contains comparisons when spectra are shifted to HDL 1b_1_, LDL 1b_1_, or HDL and LDL 1b_1_ separately.

**Figure 3:**
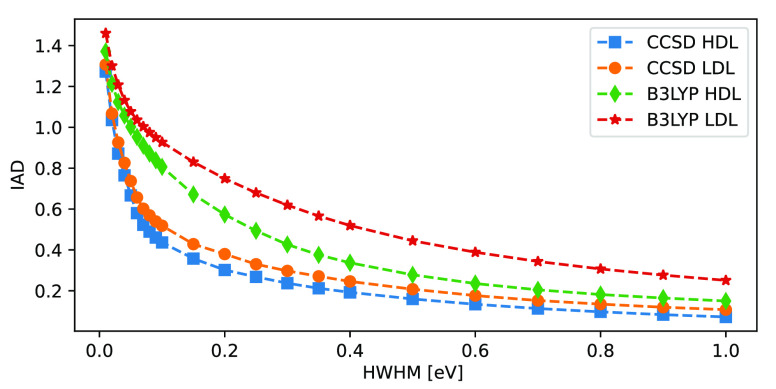
Integrated absolute difference (IAD) of EOM-CCSD and B3LYP, as calculated with a broadening parameter (half-width at half-maximum, HWHM) ranging from 0.01 to 1.00 eV, and when compared to ADC(3) reference calculations. Considering 10 HDL and 10 LDL structures and using ADC(3) as a reference.

The dependence of the IAD on the broadening parameter (HWHM) is shown in Figure 3, considering results obtained with EOM-CCSD and B3LYP. For a small HWHM, the IADs rapidly approach larger values, with an upper limit of 2. Increasing the broadening leads to decreasing IADs, approaching 0 as the peak width goes to infinity. Comparing the IAD values of the different structures and methods, the trends are similar but the relative values depend on the precise broadening value. As such, it is important to note that both the absolute and relative IADs are functions of the broadening parameter, and thus need to be clearly specified for any comparisons. In the remainder of this study we use a HWHM of 0.3 eV, which should give reasonable absolute and relative IADs.

### The Impact of HF Exchange

**Figure 4:**
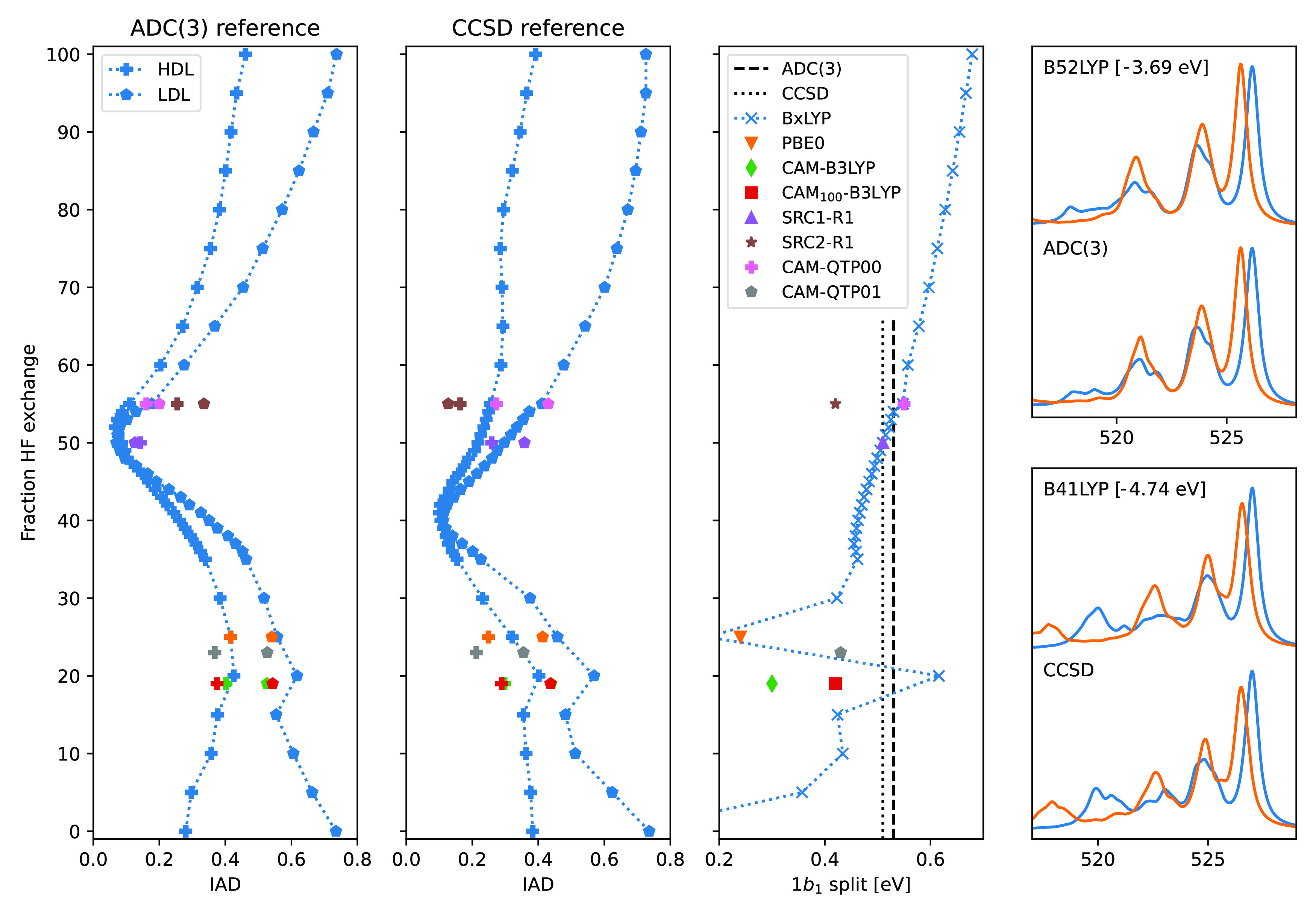
Integrated absolute difference (IAD) and 1b_1_ split for summed spectra of 10 HDL and 10 LDL clusters, as obtained using a number of xc-functionals and compared to ADC(3) and CCSD reference spectra. Functionals organized according to the fraction of short-range HF exchange. Right-most panels show the spectra of the tweaked BxLYP xc-functionals with the lowest mean IAD for ADC(3) (top) and CCSD (bottom), respectively, considering the average HDL (blue) and LDL (orange) spectra.

Using IADs and the 1b_1_ split as metrics for benchmarking TDDFT xc-functionals, Figure 4 shows the IADs when using ADC(3) or EOM-CCSD references, the 1b_1_ split, and resulting spectra for the “best” performing xc-functionals when compared to each reference separately. A number of different xc-functionals have been used, and the resulting IADs and 1b_1_ splits are ordered by the fraction of short-range HF exchange. Applied functionals include two examples from the SRC and QTP families, PBE0, B3LYP, and CAM-B3LYP with both default fraction of asymptotic HF exchange, and one tailored to yield 100% asymptotic HF exchange (CAM_100_-B3LYP).^106^ Finally, a version of the B3LYP xc-functional with variable HF exchange has been used, termed BxLYP. This functional consists of 0.81 LYP and 0.19 VWN correlation, and X HF, (0.92 – X) Becke, and 0.08 Slater exchange. For X > 0.92 the Slater exchange is also lowered, such that the sum of the three exchange parameters is always 1.

The IADs for the different functionals show similar trends when organized by the amount of HF exchange, with smaller variation with respect to remaining parameters or the functionals being global or range-separated. Small IADs are noted around 50% HF exchange when compared to ADC(3), or around 40% when compared to EOM-CCSD. However, looking at the 1b_1_ split the variations with respect to precise functional are more significant, ranging from 0.03 to 0.68 eV and generally increasing with larger fractions of HF exchange. Intriguingly, the fluctuation in IAD and 1b_1_ splits is largest for ∼20–30% HF exchange, i.e. the fraction used by many common xc-functionals. In the Supporting Information, we report the spectra of some of the functionals in the region of large (varying) 1b_1_ splits, where we observe that the split changes rapidly as a result of several peaks covering the high-energy region of primarily the LDL structures. The dominant peak then changes with the fraction of HF exchange, leading to sudden shifts in the observed 1b_1_ split. For larger fractions of HF exchange, the spectra show more well-defined peaks (similar to the gas phase), and the split varies more smoothly. In the Supporting Information the spectra of 5 HDL and 5 LDL structures are shown for ADC(3) and for TDDFT and GS-DFT with a functional with 40% HF exchange. It is seen that while GS-DFT yields reasonable results for the isolated water molecule, the cluster results have a large density of contributing states and too broad spectrum features in the case of the LDL-like tetrahedral structures. The more delocalized nature of the valence MOs yields such high density of states for GS-DFT, while for ADC(3) and TDDFT the use of a core-hole reference state localizes three MOs which then closely resemble the 1b_2_, 3a_1_, and 1b_1_ MOs of the free molecule. This thus leads to more molecule-like behavior in the spectra, which is broken for GS-DFT and for TDDFT with lower fractions of HF exchange.

The impact of the cluster size on spectra and 1b_1_ splits is illustrated in the Supporting Information, where we see that clusters of 4, 6, and 8 molecules yield relatively similar spectra for ADC(3), CCSD, and TDDFT with the SRC1-R1 and CAM-QTP00 xc-functionals. However, considering larger cluster size (32 molecules) when using TDDFT, changes in particular the LDL spectra are noted. Considering 10 HDL and 10 LDL structures, the 1b_1_ split is observed to increase by approximately 0.1 eV.

The impact on the IAD when shifting to the different 1b_1_ peak positions is illustrated in the Supporting Information, including cases where the spectra are shifted to overlap for HDL 1b_1_, LDL 1b_1_, and when calculating the IAD of LDL and HDL using separate energy shifts. We find that the difference in IAD between the HDL and LDL clusters is smaller when using separate shifts, which is to be expected. However, the IAD of HDL is generally smaller, even when both the HDL and LDL spectra are shifted to overlap LDL.

Using the IAD as the metric for identifying suitable xc-functionals for XES calculations of water clusters, the smallest IADs are obtained when using 52% HF exchange when using AD(3) as the reference and 41% HF exchange when using a CCSD reference. These functionals are here termed B52LYP and B41LYP, and we propose that these functionals are suitable for the TDDFT cluster calculations of liquid water. Using functionals that yield absolute energies close to experimental values (requiring ∼70% short-range HF exchange) is likely to produce spectra of different qualities for asymmetric and tetrahedral structures. We stress that the functionals identified here are not necessarily expected to give similar reliabilities in relative features for other elements or even for other solutions. Care should always be taken when using TDDFT, and several xc-functionals should be compared. However, the approach applied here, using IAD as a measure of relative reliability, is likely to work for other systems and edges.

In Table 1 we report the 1b_1_ energy split of ADC(3), CCSD, and TDDFT with 10 different xc-functionals, as well as the energy splits and IADs compared to ADC(3) or CCSD reference spectra. Included are TDDFT results obtained using four customized functionals (B3LYP and BHandHLYP with tailored fractions of HF exchange), B3LYP, CAM-B3LYP, two functionals from the QTP family, and two from the SRC family. The B50LYP* and B40LYP* functionals are constructed in similar manners as B52LYP and B41LYP, but using BHandHLYP as the base instead of B3LYP—this yields slightly different fractions of HF exchange but only minor differences in 1b_1_ splits and IADs.

**Table 1:** Statistics for X-ray Emission Spectrum Calculations of 10 HDL and 10 LDL Structures^a^

		Versus ADC(3)			Versus CCSD		
Method	1b_1_ split	Δ*E*	HDL IAD	LDL IAD	Δ*E*	HDL IAD	LDL IAD
ADC(3)	0.53	–	–	–	0.85	0.24	0.30
CCSD	0.51	–0.85	0.24	0.30	–	–	–
B52LYP	0.52	–3.69	0.07	0.08	–2.84	0.24	0.34
B41LYP	0.46	–5.59	0.24	0.33	–4.74	0.10	0.11
B50LYP*	0.51	–4.38	0.07	0.06	–3.53	0.22	0.32
B40LYP*	0.47	–6.11	0.24	0.33	–5.26	0.10	0.11
B3LYP	0.62	–8.80	0.43	0.62	–7.95	0.40	0.57
CAM-B3LYP	0.30	–9.19	0.40	0.53	–8.34	0.30	0.44
SRC1-R1	0.51	–4.08	0.14	0.13	–3.23	0.26	0.36
SRC2-R1	0.42	–6.34	0.25	0.34	–5.49	0.16	0.13
CAM-QTP00	0.55	–3.38	0.16	0.20	–2.53	0.27	0.43
CAM-QTP01	0.43	–8.06	0.37	0.53	–7.21	0.21	0.36

a Including 1b_1_ energy splits, energy alignment (Δ*E*) for calculations of integrated absolute differences (IADs), and IADs with ADC(3) or CCSD as a reference. Considering ADC(3), CCSD, and TDDFT calculations with 10 xc-functionals. Energies in eV.

The 1b_1_ split is seen to vary from 0.30 to 0.62 eV, with those of the reference methods amounting to 0.53 and 0.51 eV for ADC(3) and CCSD, respectively. The largest split is seen to be for B3LYP, but adding long-range correction (CAM-B3LYP) changes this to a very small shift, and the IADs are in both cases in the upper range (0.30–0.43), indicating that these functionals are not suitable. The reason for the sudden shift is due to a high density of states in the 1b_1_ region, as illustrated in SI. The IAD when comparing ADC(3) and CCSD amounts to 0.24 and 0.30 for HDL and LDL, respectively. This difference, as well as the errors in absolute energies (see comparison to experiment in Figure 1) reflects missing relaxation and other deficiencies in the calculations. However, using the 1b_1_ split and IAD as metrics of the performance of xc-functionals for calculating X-ray emission spectra of water clusters, we posit that the four customized functionals, SRC1-R1, and CAM-QTP-00 yield reasonable relative spectra. As the ADC(3) results have more defined features (similar to experiments), we deem this method to be slightly more suitable for these calculations, and the tailored B52LYP is proposed to provide a reasonable balance between accuracy and computational cost.

## Conclusions

The X-ray emission spectrum of liquid water possesses a split in the high-energy 1b_1_ peak, for which the origin is still debated. For reliable spectrum calculations, it is vital to be able to both produce highly reliable structures and dynamics, as well as X-ray spectra of high quality. Here we investigate the performance of TDDFT for calculating X-ray emission spectra, using highly reliable ADC(3) and CCSD results as reference. It is noted that X-ray emission calculations using ADC(2) and ADC(2)-x (which perform very well for isolated molecules^54,55^) are unsuitable, due to mixing with satellite states of incorrect energies. Using the integrated absolute difference (IAD)^72−75^ as a metric, it is clear that the simultaneous reproduction of spectra for highly asymmetric (high density liquid, HDL) and tetrahedral (low density liquid, LDL) clusters is challenging, with the amount of HF exchange in the exchange-correlation functional highly impacting the absolute and relative IADs as well as the obtained split in the 1b_1_ region. The IAD is calculated from the difference between shifted and area-normalized spectra and thus measures agreement in both relative energies and intensities.

Tailored B3LYP functionals with higher fractions of HF exchange are seen to yield good agreement with the reference methods, with 52% being favored when compared to ADC(3), and 41% when compared to CCSD. These functionals also yield 1b_1_ energy splits in good agreement with the reference methods, resulting in HDL clusters showing a peak ∼0.5 eV above that of the LDL clusters. The experimental split is ∼0.8 eV, but we stress that a direct comparison to our results is unsuitable, as our calculations have been carried out for relatively small clusters without including core-hole-induced proton dynamics and featuring distinctly asymmetric or tetrahedral structures. For example, using larger structures of totally 32 water molecules is here seen to lead to an increase in 1b_1_ energy splits by ∼0.1 eV, as observed when using TDDFT.

The integrated absolute difference (IAD) provides a metric that is suitable when dealing with ensembles of structures and investigating the performance of several methods using different reference methods simultaneously. It provides a single value that agrees well with visual comparisons of spectra as it focuses on relative energies and intensities, i.e. overall similarity of spectra. This is particularly interesting for studies in the X-ray regime, where, in particular, absolute energies can be difficult to pinpoint with good accuracy (and good absolute energies do not necessarily correspond to good relative features^82^). Some care should be taken when aligning spectra, and the resulting values will depend on the energy shift, the energy region that is integrated over, and the broadening scheme. Nevertheless, we believe that this measure is highly suitable for comparative studies of ensembles of structures and encourage further adaptation and evaluation.

Based on the present study, we finally conclude that the here defined B52LYP and B41LYP functionals provide a balanced description of XES spectra of water molecules in both the tetrahedral and disordered local coordination with respect to agreement with higher-level reference methods (ADC(3) and CCSD) in terms of both spectral shape and 1b_1_ split.

## Supporting Information Available

Additional calculations: IAD and 1b_1_ for different numbers of structures, gas phase and hexamer spectra of the ADC hierarchy, spectra of individual structures, spectra using select xc-functionals, spectra from GS-DFT, the impact of cluster size, IADs when using different energy shifts, description of how oxygen ECPs were included, and geometries of isolated molecule and 20 six-molecule clusters (PDF)
