# Supplementary material for: Calibrating TDDFT Calculations of the X-ray Emission Spectrum of Liquid Water: The Effects of Hartree–Fock Exchange
Source: J Chem Theory Comput. 2023 Oct 3;19(20):7333–42. doi: 10.1021/acs.jctc.3c00728 (PMC10601479; doi:10.1021/acs.jctc.3c00728)
Supplement: Supplementary file 1 — ct3c00728_si_001.pdf [file ct3c00728_si_001.pdf]

Supporting information for "Calibrating TDDFT calculations of the X-ray emission spectrum of liquid water: the effects of Hartree-Fock exchange"

Thomas Fransson and Lars G. M. Pettersson*
Department of Physics, AlbaNova University Center, Stockholm University
109 61 Stockholm, Sweden

Impact of the number of structures

The integrated absolute difference (IAD) and $1b_1$ split when using different numbers of structures (n) are shown in Fig. S1. Relatively stable IADs and $1b_1$ splits are seen for n>5, and n=10 is thus deemed to be sufficient for the purposes of this study. The ADC(3) spectra of the 10 individual clusters are shown in Fig. S2, together with the averaged spectra.

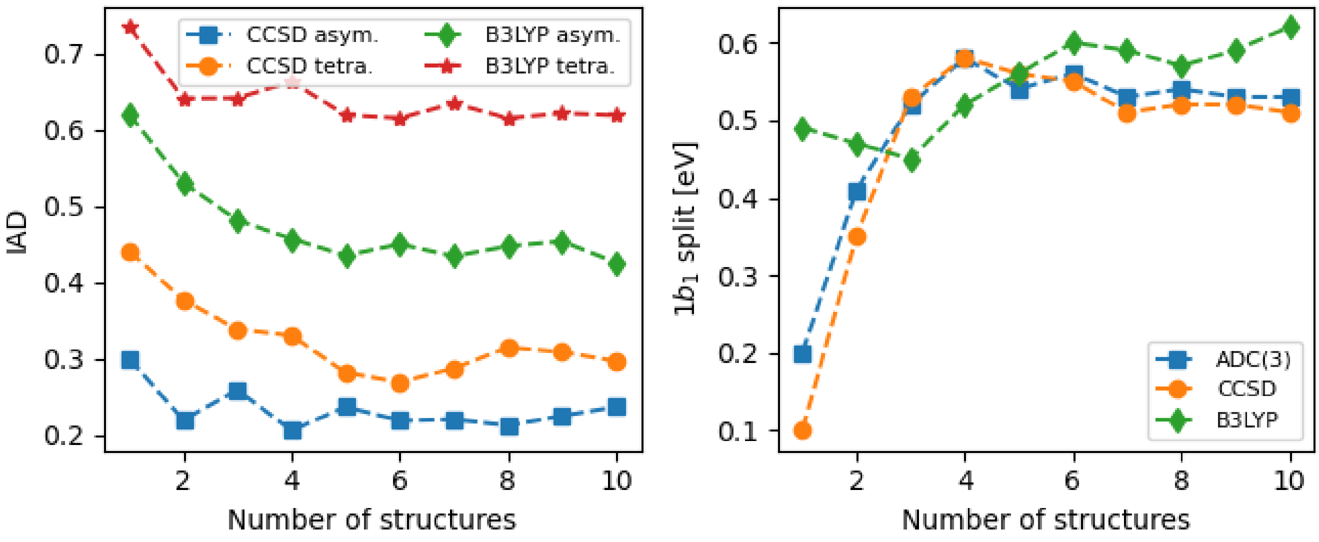

Fig. S1: Left: IAD of CCSD and TDDFT (B3LYP) with reference to ADC(3), considering varying number of structures. Right: Peak split as a function of number of structures.

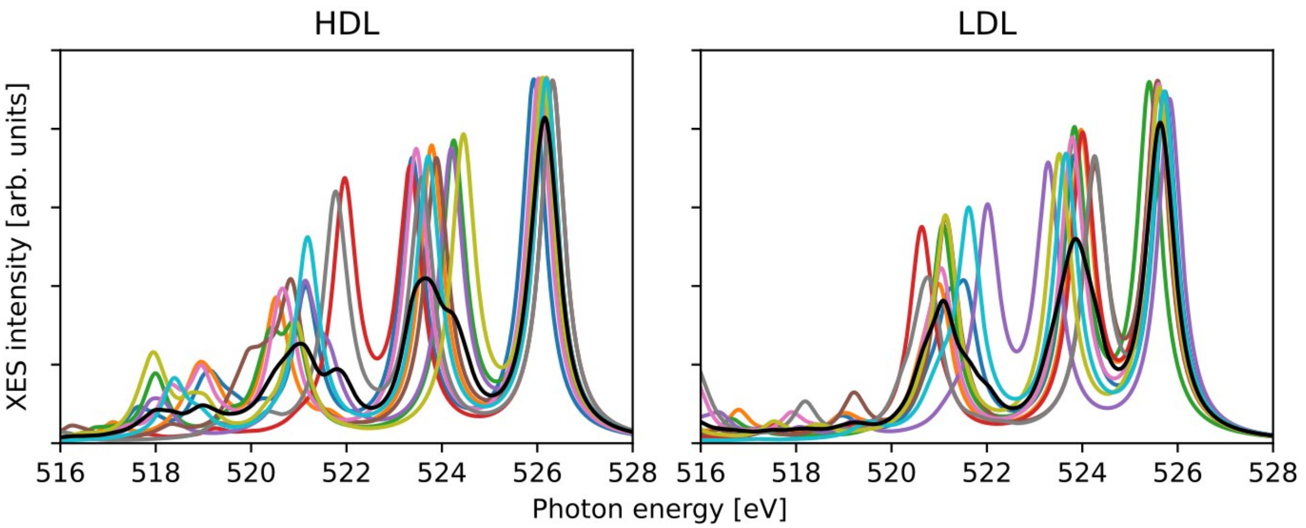

Fig. S2: Computed X-ray emission spectra of 10 HDL and 10 LDL structures, as obtained using ADC(3). Individual spectra as well as the average (thick black line) are shown.

Performance of the ADC hierarchy

In Fig. S3 the spectra of a single molecule and a single hexamer are shown, as calculated with ADC(2), ADC(2)-x, and ADC(3). The gas phase spectra are similar in terms of relative features, density of states, and amount of single-excitation amplitudes (the single-excitation character of a state; $|v1|^2=1$ for a pure single-excitation). However, for the hexamers the ADC(2) and ADC(2)-x have a higher number of states, in particular in the $3a_1$ and $1b_1$ region, the intensity is distributed over a wider region, and the states have a lower amount of single-excitation amplitudes. This is due to doubly-excited states mixing in with the singly-excited states for ADC(2) and ADC(2)-x, while for ADC(3) the improved description of double-excitations lead to them appearing at different energies.

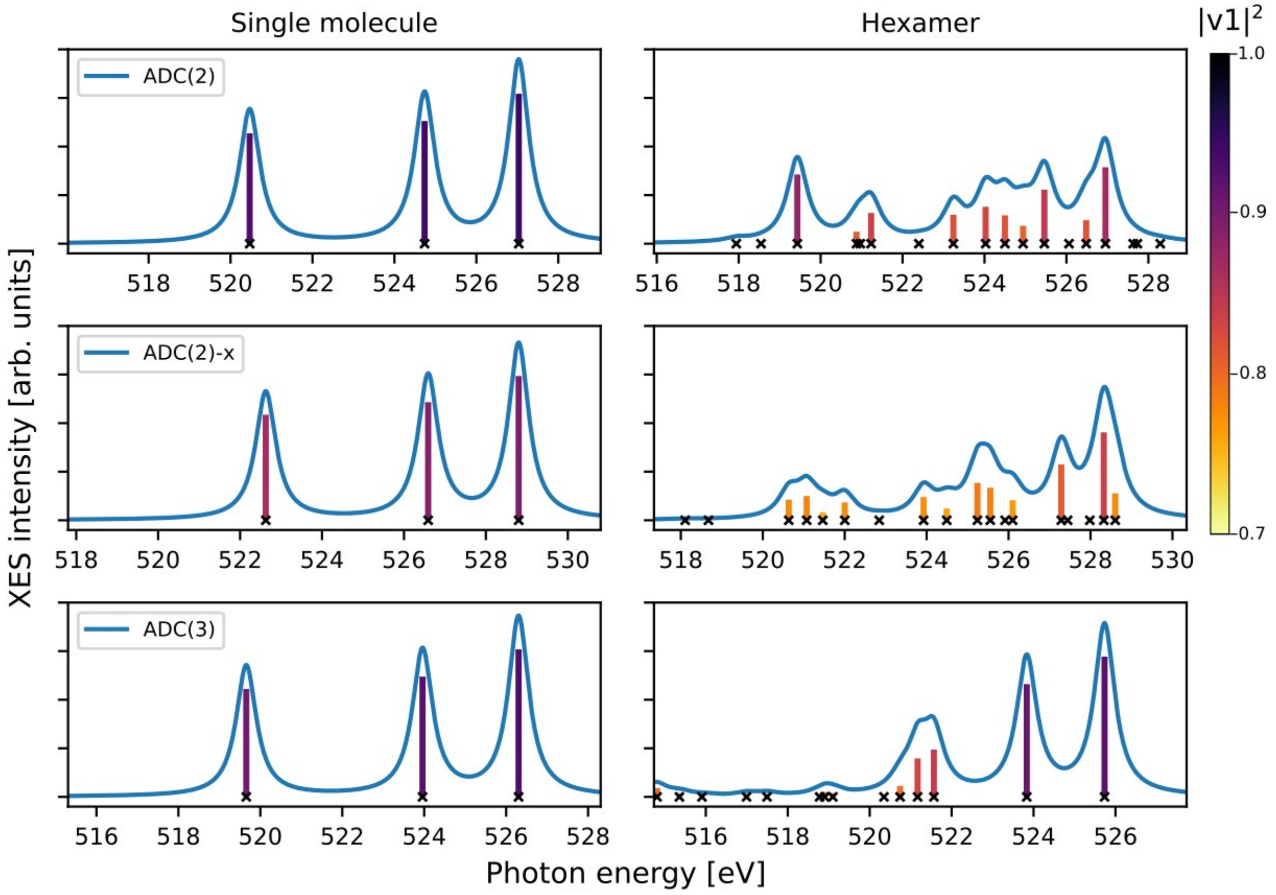

Fig. S3: X-ray emission spectra of the isolated water molecule and a six-molecule cluster, as calculated with ADC(2), ADC(2)-x, and ADC(3). Position of each state is marked with a black cross, and bar spectra are included where the bars are colored by the single-excitation amplitude character ($|v1|^2$) of each state.

TDDFT spectra of selected functionals

Fig. S4 shows the HDL and LDL spectra for six different xc-functionals, in particular from regions where the fraction of HF exchange yields large changes in IAD and $1b_1$ split. Comparing results using BxLYP with 20 and 25% HF exchange, the split increases as a result of a shift in which LDL peak in the $1b_1$ is most intense. Increasing the exchange to 30% changes the features to be somewhat more defined (for the LDL clusters), with a $1b_1$ split larger than for 25%. Further increasing the fraction of HF exchange makes the features more well-defined, and the $1b_1$ split varies smoothly. Instead increasing the amount of long-range HF exchange (*i.e.* using CAM-B3LYP) does not lead to more defined features, but the relative intensities of the states change and the $1b_1$ split is seen to decrease from 0.62 eV (B3LYP) to 0.30 eV (CAM-B3LYP).

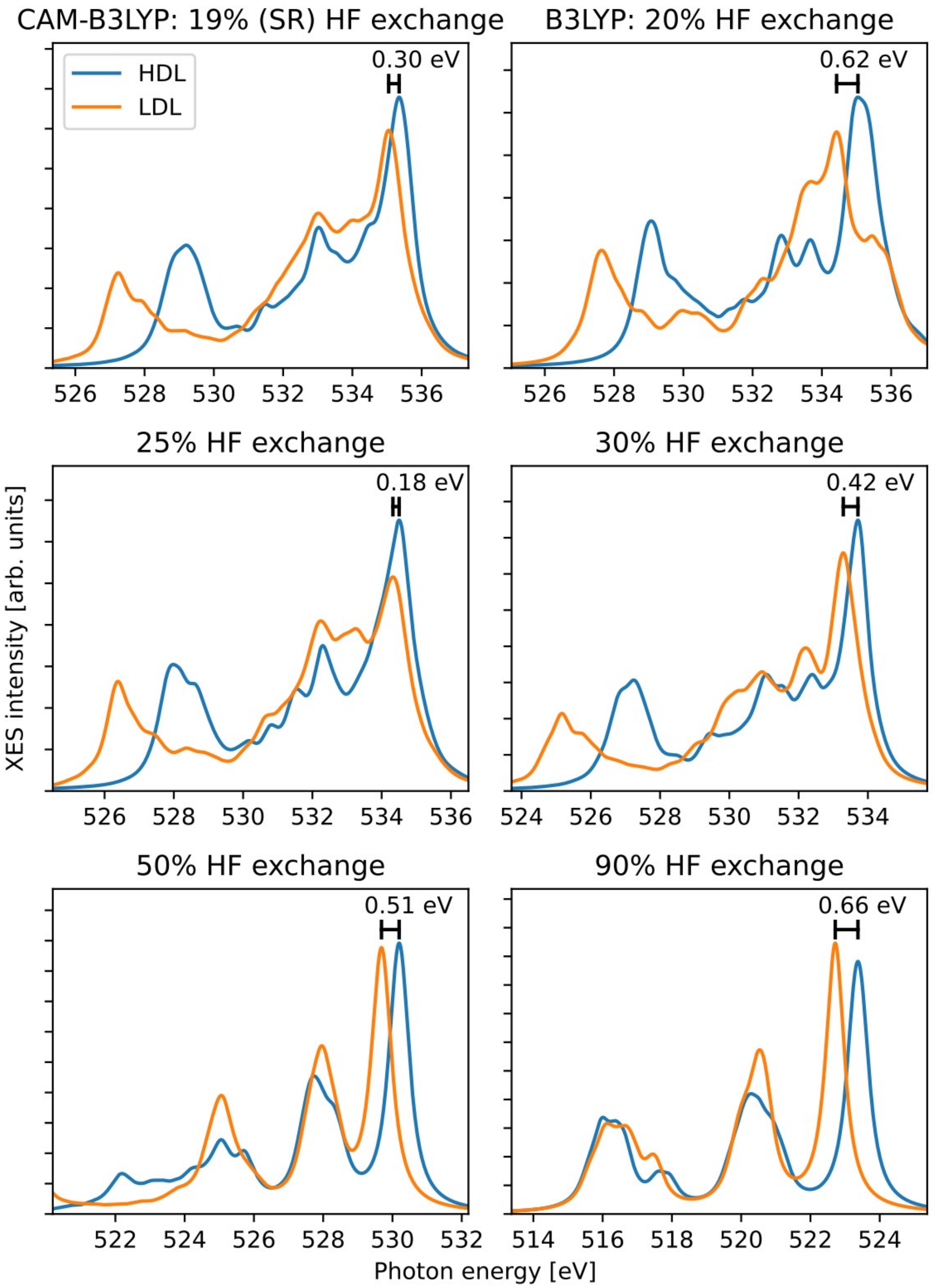

Fig. S4: Summed X-ray emission spectra of 10 HDL and 10 LDL water clusters, as obtained using TDDFT with six different exchange-correlation functionals. Including the peak separation between the $1b_1$ features, *i.e.* the $1b_1$ split.

Spectra from GS-DFT

The X-ray emission spectra obtained using ADC(3) and TDDFT and GS-DFT are shown in Fig. S5, utilizing tailored BxLYP functionals with 40% HF exchange. Results are shown for the gas phase and for 5 HDL and 5 LDL clusters. For the gas phase the TDDFT and ADC(3) spectra are very similar, while the GS-DFT features are

a bit more compressed (in particular the relative position of $1b_2$). The experimental energy difference between $1b_1$ and $1b_2$ is ~6.1 eV, and we obtain shifts of 5.8, 6.6, and 6.7 eV for GS-DFT, TDDFT, and ADC(3), respectively.

For the hexamer clusters, the differences in the spectra are more pronounced, with the TDDFT and ADC(3) results being relatively similar (at least in the region of the $1b_1$ peak), but the GS-DFT results in a significantly broadened spectrum, in particular for the LDL clusters. The broader GS-DFT features result from relatively delocalized molecular orbitals, which leads to intensity being distributed over a wider energy region. By comparison, using a two-step approach where a core-hole reference state is formed, MOs are more localized to the individual water molecule, and thus take a more "molecule"-like behavior. For calculations on individual molecules or singly-hydrogen-bonded systems this delocalization is less of an issue, and GS-DFT has thus been successfully applied for such systems. However, when considering solutions the GS-DFT approach may not be suitable, and explicit core-hole calculations with accompanying MO localizations are recommended.

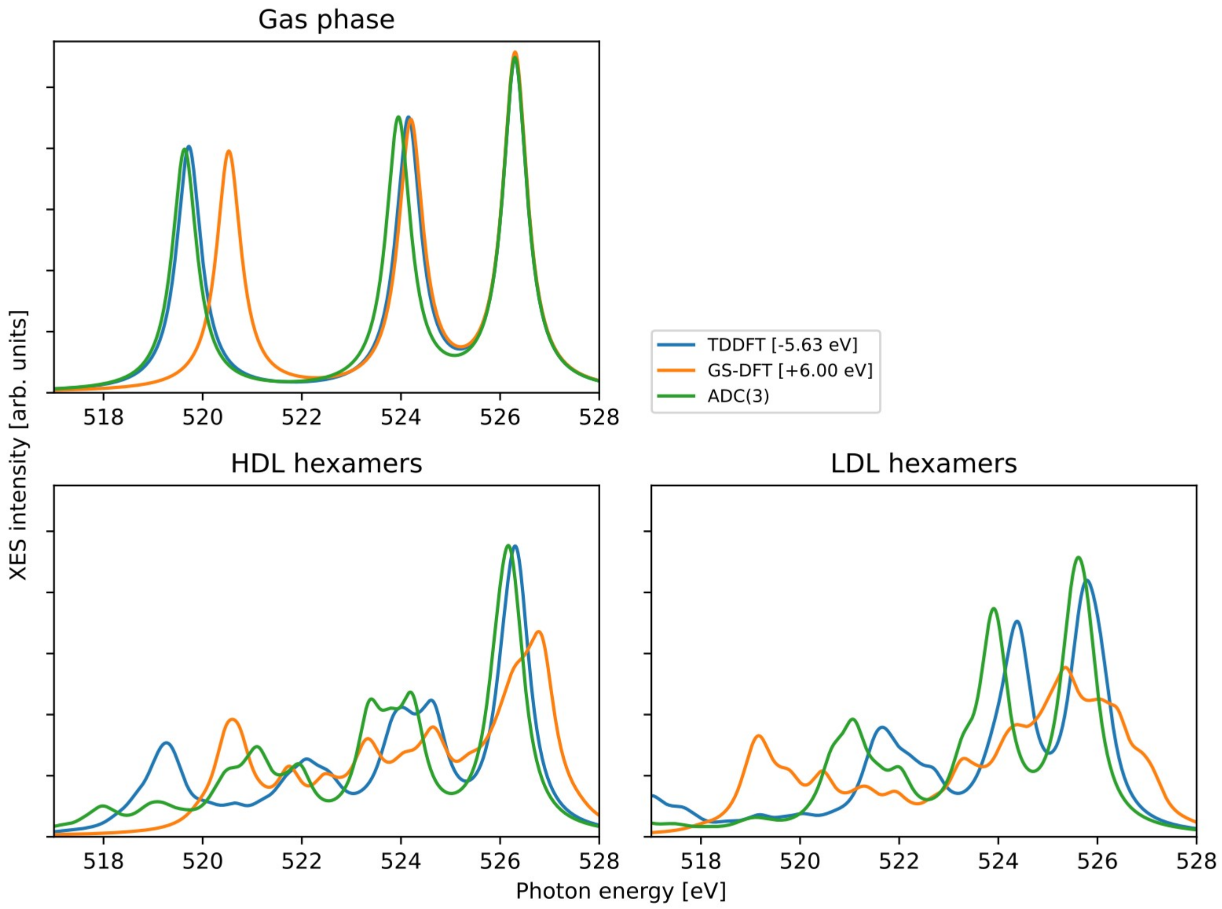

Fig. S5: X-ray emission spectra of an isolated water molecule, and summed spectra of 5 HDL and 5 LDL clusters, as computed using ADC(3) and TDDFT/GS-DFT with a BxLYP functional with 40% HF exchange. Results are aligned to $1b_1$ of ADC(3).

Impact of the size of the clusters

The ADC(3) and CCSD spectra when using clusters with 4, 6, and 8 molecules are shown in Fig. S6. The results obtained using 4 and 8 molecules are compared to that of 6 molecules, and only relatively small differences are visible, primarily present for the smallest structure.

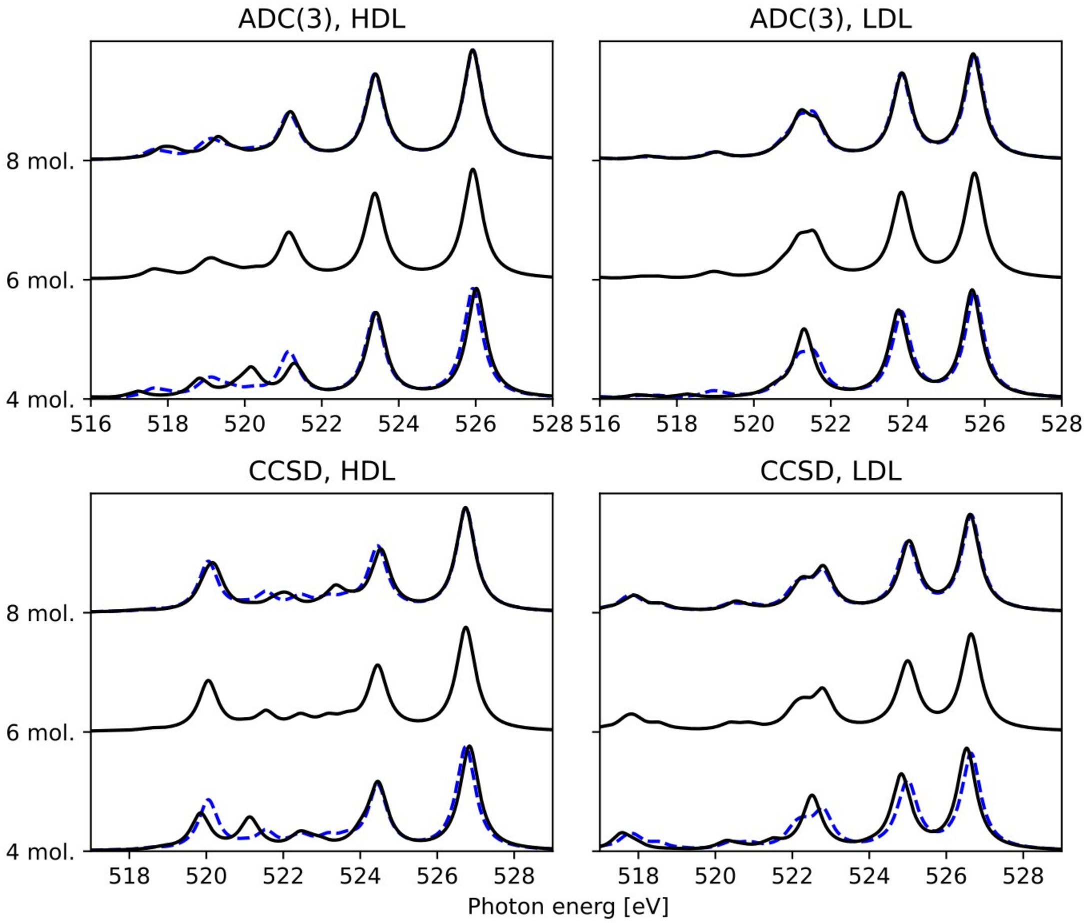

Fig. S6: X-ray emission spectra of one asymmetric (HDL) and one tetrahedral (LDL) cluster, using ADC(3) and CCSD and considering different cluster sizes. Black lines showing spectra of the current cluster size, and dashed blue line shows the results using 6 molecules.

Fig. S7 illustrates the impact of cluster size when using TDDFT, considering the SRC2-R1 and CAM-QTP00 xc-functional. Here, we note small differences between the structures with 4, 6, and 8 molecules (in particular between 6 and 8), but a more significant blue-shift when including 32 molecules. This shift is different for the HDL and LDL structures, and we show the results when considering ten different structures with 6 or 32 molecules. A consistent blue-shift is noted, featuring ~0.08 eV for HDL structures, and ~0.18 eV for LDL structures. This indicates an increase in the $1b_1$ split of ~0.1 eV for larger clusters, at least for TDDFT and when considering highly asymmetric HDL and tetrahedral LDL structures. As such, we suggest that an increase in the $1b_1$ split of 0.1 eV may be an upper limit when moving from small to large clusters.

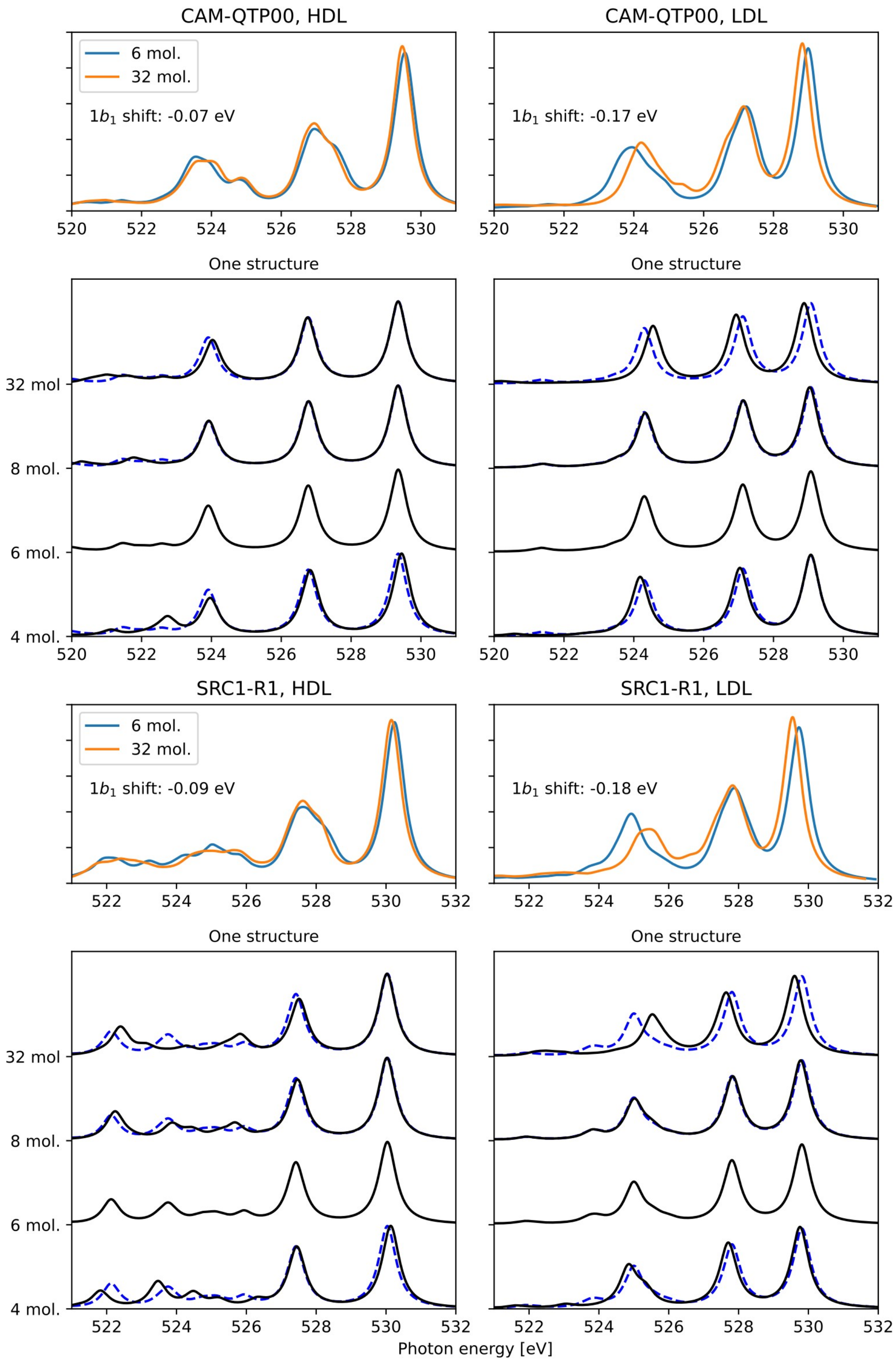

Fig. S7: X-ray emission spectra of 1 or 10 HDL and LDL structures, obtained using TDDFT with two different xc-functionals. For comparisons using one structure, spectra using different cluster sizes (black line) are compared to results obtained when using six water molecules (dashed blue line). For the ensembles of 10 structures we report the shift in $1b_1$ energies.

IAD when shifting to different $1b_1$ peaks

In Fig. S8 we illustrate the integrated absolute difference (IAD) trends when shifting spectra to different features before calculating the IAD. Left panel shows results when shifting to the HDL $1b_1$ (as used throughout this work), and the middle panel when shifted to the LDL $1b_1$. Finally, in the right panel we report the results when shifting HDL and LDL spectra individually. We see that the trends are very similar, with the main differences being in the relative IADs for high fractions of HF exchange, as well as some of the fluctuations for low levels of HF exchange. The IAD minima are relatively unaffected by the precise shift in use, and we consider the present choice of an HDL reference to be appropriate.

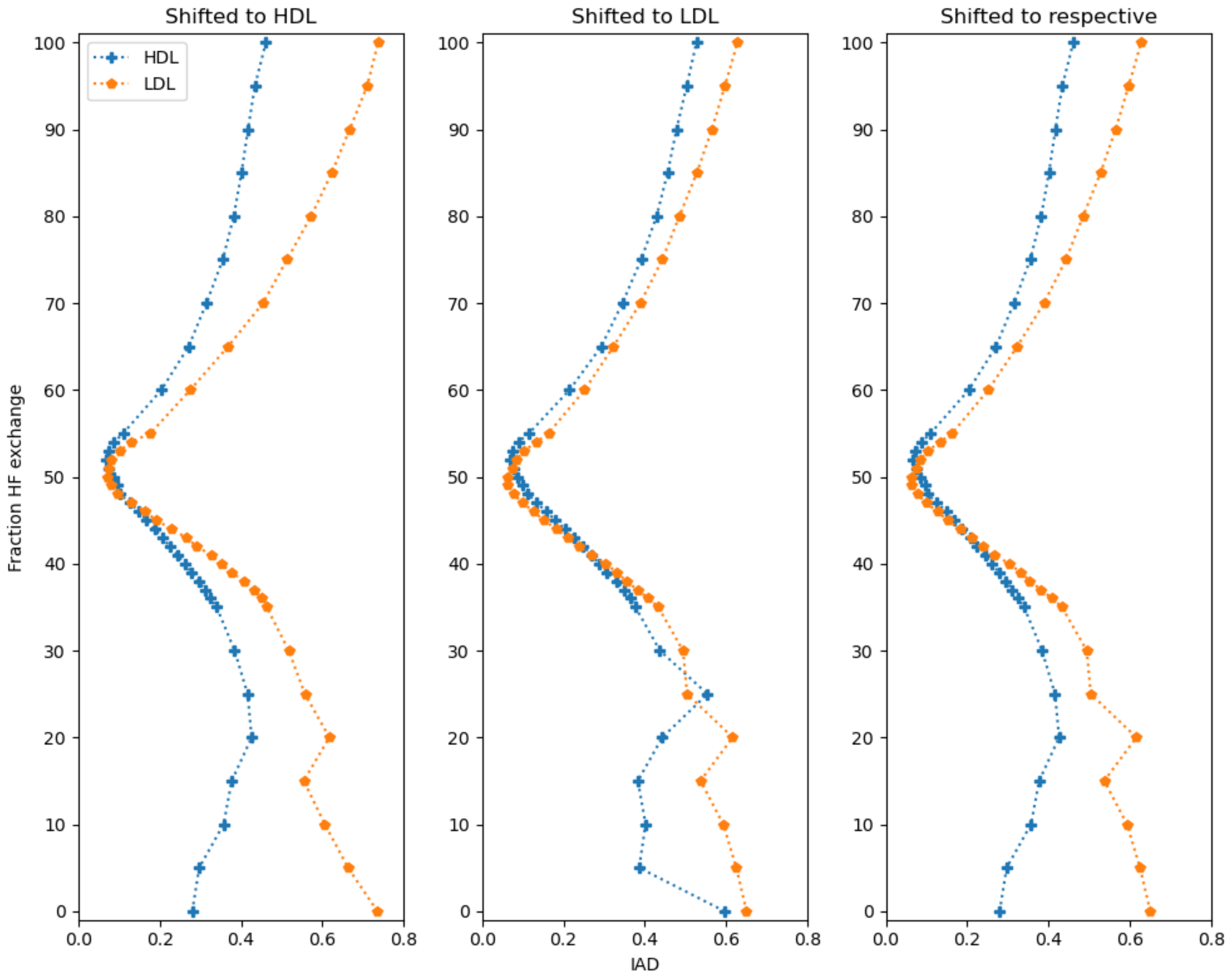

Fig. S8: Integrated absolute differences (IADs) of X-ray emission spectra calculated using TDDFT with BxLYP, and calculated when comparing to ADC(3) results. Showing trends when shifting BxLYP spectra to align with ADC(3) for the HDL $1b_1$ (left), LDL $1b_1$ (middle), and with the HDL and LDL features shifted individually (right).

Including effective core potentials on selected oxygens

Q-Chem does not allow different descriptions (all-electron or ECP) for different atoms of the same element. Thus, we performed the present calculations by assigning the surrounding oxygens as Neon atoms, but represented with an Oxygen basis set and an Oxygen effective core-potential (ECP) called Neon, but with four electrons removed to give the effective nuclear charge of an Oxygen atom, rather than removing two as would be done for Neon. This is fully consistent with an ECP description of Oxygen and eliminated the 1*s* core levels of all oxygens except for the selected central water molecule, thus making its 1*s* state unique.

Geometries

Geometries of the isolated water molecule and of 10 asymmetric (HDL) and 10 tetrahedral hexamers are given below, as expressed in Ångström. The first oxygen is the central, probed, oxygen.

Isolated molecule:

O	0.000000000	0.000000000	0.118729000
H	-0.753201000	0.000000000	-0.474916000
H	0.753201000	0.000000000	-0.474916000

HDL1:

O	0.000000	0.000000	0.000000
O	-1.121700	1.020400	2.369100
O	-0.489000	-2.768800	-0.659300
O	-0.661300	2.791800	-0.793100
O	1.091210	0.414400	-3.064300
O	2.465590	-2.461700	-0.943700
H	-0.199900	0.302500	-0.854800
H	-0.075900	-0.905300	-0.220300
H	-1.997900	0.405300	2.461300
H	-0.684700	0.703300	1.609000
H	-1.209700	-3.056900	-1.445500
H	0.357700	-3.081700	-1.035100
H	-1.320600	2.820700	-0.090200
H	0.217500	3.057100	-0.626600
H	1.436060	0.876400	-3.970800
H	1.663640	0.828100	-2.342300
H	2.463120	-1.781000	-1.571000
H	2.837190	-2.109700	-0.060000

HDL2

O	0.000000	0.000000	0.000000
O	-2.337100	-0.867200	-1.325500
O	1.582100	2.378800	0.278600
O	-0.286200	-0.426900	2.946100
O	-2.790800	0.537600	1.607000
O	-0.586200	-2.992500	-1.361200
H	0.687900	-0.712700	0.343700
H	0.436100	0.785200	0.138700
H	-1.634100	-0.653600	-0.837100
H	-2.837300	-1.170100	-0.676400
H	2.143500	2.312400	-0.532200
H	1.416000	3.326800	0.123400
H	-0.623400	0.027900	2.372600
H	0.033300	-1.075000	2.483900
H	-3.017100	1.046900	0.813700
H	-3.439500	-0.244500	1.637900
H	-1.459100	-2.546500	-1.339300
H	-0.141100	-3.063800	-0.460000

HDL3:

O	0.000000	0.000000	0.000000
O	2.350800	-1.428500	-0.592700
O	-2.292700	-1.543900	0.822700
O	-1.338500	2.653000	-0.838800
O	-1.023000	-1.730000	-2.624400
O	2.384000	0.516100	-2.718700
H	0.130800	0.841200	0.593500
H	-0.768700	-0.522400	0.329300
H	2.119300	-2.549000	-0.458700
H	1.391900	-0.993600	-0.407900
H	-2.215700	-1.590200	1.821200
H	-1.878700	-2.409900	0.559600
H	-0.906300	1.733690	-1.304100
H	-2.303900	2.558060	-0.786300
H	-1.113800	-1.330900	-3.539900
H	-1.266800	-0.855500	-2.157100
H	2.455300	0.513200	-3.636400
H	2.226500	-0.561400	-2.322300

HDL4:

O	0.000000	0.000000	0.000000
O	-1.618500	-1.961090	-1.291100
O	2.643800	-0.262230	1.144400
O	-0.871700	3.068110	-0.379900
O	-3.262900	0.693770	0.752100
O	1.130600	2.375210	-2.395100
H	0.961600	-0.129300	0.490000
H	-0.015400	0.885270	0.518400
H	-1.152500	-1.147590	-0.908000
H	-1.380200	-1.970790	-2.244600
H	2.903600	0.310130	0.507200
H	3.059300	-0.074390	2.030400
H	-1.500600	3.647810	-0.779400
H	-1.199200	2.933810	0.577000
H	-3.022300	0.716030	-0.094600
H	-2.952900	-0.208730	1.092100
H	0.509400	2.743010	-1.670700
H	1.579100	3.184710	-2.635300

HDL5:

O	0.000000	0.000000	0.000000
O	0.233900	1.967800	2.016882
O	0.444600	-2.816600	-0.112128
O	-2.369270	1.743600	0.723932
O	-0.379900	-1.285510	2.972482
O	-0.127800	-0.232600	-3.318318
H	0.708700	0.356700	-0.772078
H	0.161100	-1.008810	0.021776
H	0.188000	1.442200	1.340882
H	-0.176000	2.919800	1.625582
H	1.160800	-2.770970	-0.745378
H	-0.485500	-2.680240	-0.594048
H	-3.156280	1.477900	1.113882
H	-2.125250	1.164200	-0.009698
H	-0.781100	-0.402600	3.432282
H	0.503100	-1.180430	2.736882
H	0.545800	-0.223800	-2.742418
H	-1.044300	-0.071700	-2.842918

HDL6:

O	0.000000	0.000000	0.000000
O	-1.918300	1.900700	0.815000
O	0.411000	-1.052300	-2.619300
O	1.418300	1.820000	1.982000
O	-2.052400	1.048400	-2.070800
O	0.612600	2.570800	-1.961200
H	0.011500	-0.368300	-0.915400
H	0.615200	-0.630900	0.431600
H	-1.306700	1.148500	0.595800
H	-2.612700	1.793800	0.112800
H	0.089200	-0.692800	-3.461470
H	0.486700	-2.019100	-2.760800
H	1.119600	2.729500	2.145100
H	2.070100	1.845500	1.252400
H	-1.170500	1.458900	-2.110100
H	-2.049400	0.634800	-2.956700
H	1.101000	1.930500	-1.467500
H	0.322500	3.274800	-1.355400

HDL7:

O	0.000000	0.000000	0.000000
O	-1.908500	2.005800	-0.591500
O	-1.642000	-2.294600	0.477900
O	1.676100	1.822300	1.845400
O	-0.105800	-0.706200	-3.201500
O	2.489500	-1.223800	-1.988600
H	-0.416600	-0.791300	0.119700
H	0.665400	-0.090200	0.688500
H	-1.251000	1.412100	-0.238000
H	-2.285700	2.284100	0.065000
H	-1.523900	-2.332800	-0.500800
H	-2.638300	-2.273400	0.480400
H	2.117500	2.152900	0.970700
H	1.984200	2.571100	2.394800
H	-0.044900	0.136800	-2.889600
H	-0.989500	-1.164200	-2.741000
H	1.617500	-0.882500	-1.819400
H	2.328900	-1.517000	-2.925000

HDL8:

O	0.000000	0.000000	0.000000
O	2.126800	-1.187000	1.505400
O	-1.882600	2.051600	0.686300
O	1.283400	2.056400	1.937800
O	-3.111700	-0.625850	0.184800
O	-1.823800	-0.976990	-2.738400
H	0.816700	-0.361900	0.492900
H	0.099700	-0.812230	-0.718000
H	2.924600	-1.840430	1.218200
H	2.127900	-1.130590	2.492600
H	-1.289000	1.217500	0.454800
H	-1.319900	2.789000	0.955500
H	1.337000	2.226500	0.953900
H	2.230200	1.911500	2.161300
H	-3.914600	-0.647250	-0.352000
H	-2.820200	-1.547654	0.318900
H	-2.407900	-0.672350	-2.095000
H	-1.660300	-1.835190	-2.755200

HDL9:

O	0.000000	0.000000	0.000000
O	2.748740	0.464400	-0.379600
O	-0.303840	-1.516546	-2.395800
O	-0.164260	2.973200	0.753800
O	-1.891660	2.309600	-1.289300
O	-0.234660	1.420200	-3.050900
H	-0.552760	-0.194400	0.886400
H	-0.146080	-0.624400	-0.706200
H	3.435140	-0.086000	0.254900
H	1.796640	0.335600	-0.057100
H	-1.193160	-1.639130	-2.428800
H	-0.018190	-0.735640	-2.972600
H	-0.209530	2.199500	1.408100
H	-0.627760	2.468000	0.094500
H	-2.309560	1.410900	-1.118400
H	-2.610760	3.002800	-1.220900
H	-0.376730	2.230700	-2.730900
H	0.779490	1.669900	-3.281100

HDL10:

O	0.000000	0.000000	0.000000
O	-1.132400	-1.740500	1.924800
O	0.878870	2.635700	-0.648800
O	0.325600	2.199540	2.091900
O	3.041800	-0.557600	-0.925800
O	2.748300	-1.629500	1.569300
H	-0.295700	-0.171000	-0.969800
H	0.392800	0.903490	-0.144600
H	-0.391300	-2.617900	2.002300
H	-0.840600	-1.195400	1.275200
H	0.921890	2.745400	0.380500
H	1.801060	2.346500	-0.796800
H	0.703620	2.583400	2.837700
H	0.444100	1.195790	2.317800
H	3.922200	0.025600	-1.106000
H	2.975300	-0.619500	0.069600
H	2.023010	-2.171100	1.863000
H	2.951800	-1.140100	2.485600

LDL1:

O	0.000000	0.000000	0.000000
O	-1.991300	-1.410500	0.452660
O	-0.597900	1.005200	-2.324880
O	0.734600	1.507600	2.021820
O	1.934700	-1.690300	-0.684180
O	0.689400	-0.854700	3.565820
H	0.003300	0.357900	-0.933880
H	0.409300	0.750900	0.540590
H	-1.482400	-2.166700	0.742660
H	-1.244200	-0.872900	0.213830
H	-0.506200	0.433100	-3.103380
H	-1.502200	1.329000	-2.345080
H	-0.010700	2.112800	2.105920
H	1.479300	1.955200	2.385120
H	2.508800	-1.344500	-1.352380
H	1.230000	-1.012900	-0.503380
H	0.431100	-0.164000	2.965120
H	0.438700	-0.536500	4.463320

LDL2:

O	0.000000	0.000000	0.000000
O	0.117000	1.680300	1.993800
O	-0.445400	-2.492040	0.821800
O	-2.593300	-0.333300	-0.559700
O	1.011200	-0.029800	-2.480600
O	-1.880000	0.155800	-3.477500
H	0.601200	0.028400	-0.735500
H	0.264100	0.527200	0.761500
H	1.014000	1.796700	2.417600
H	-0.007600	2.556800	1.604400
H	0.211900	-3.162130	1.106500
H	-0.088000	-1.630300	0.544800
H	-3.082650	-1.037600	-0.081900
H	-1.698200	-0.248500	-0.146000
H	1.606200	-0.667000	-2.950500
H	0.156300	0.095700	-2.961300
H	-2.185600	1.083200	-3.675200
H	-2.187700	0.021700	-2.599700

LDL3:

O	0.000000	0.000000	0.000000
O	0.651570	2.374700	0.586100
O	-0.875150	-0.078300	-2.426900
O	-2.075750	-1.247800	0.956900
O	2.104950	-1.439000	0.622500
O	-1.292050	0.415300	3.573200
H	-0.288470	-0.103000	-0.888800
H	0.473272	0.895600	0.138600
H	0.325610	2.808000	-0.238500
H	0.172450	2.781300	1.284400
H	-1.775550	-0.107500	-2.283700
H	-0.758450	0.910900	-2.843600
H	-1.371350	-0.762400	0.684900
H	-1.769350	-2.175000	1.236200
H	1.440090	-1.021200	0.292300
H	1.858850	-2.281900	0.940800
H	-1.407050	-0.290500	2.835200
H	-1.558650	-0.028400	4.362600

LDL4:

O	0.000000	0.000000	0.000000
O	-1.978200	-1.150070	1.256800
O	0.473800	-0.516870	-2.567290
O	-1.352200	2.085430	-0.964500
O	2.257900	0.481890	1.344600
O	-2.235200	2.540530	1.580600
H	0.826000	0.111090	0.517000
H	-0.677300	-0.511270	0.466100
H	-2.887900	-1.025670	0.959400
H	-1.790900	-2.094670	1.439100
H	0.418400	-0.300170	-1.628600
H	0.925100	0.272570	-2.985510
H	-0.816900	2.897230	-0.945700
H	-0.755500	1.419860	-0.594600
H	2.820400	1.269930	1.378000
H	2.319700	0.081820	2.255600
H	-2.266900	1.890290	2.302800
H	-1.657900	2.225530	0.854300

LDL5:

O	0.000000	0.000000	0.000000
O	-2.350600	0.823000	0.679000
O	1.111300	-2.335800	-0.325200
O	0.862900	1.769560	1.736600
O	-0.237600	0.600000	-2.554630
O	-0.066500	3.867100	-0.049000
H	0.567200	0.719600	0.458600
H	0.092900	0.421700	-0.824500
H	-2.359200	1.466700	1.288100
H	-1.402800	0.592900	0.627300
H	0.222400	-2.717500	-0.574200
H	0.825400	-1.379900	-0.153000
H	0.873500	2.447080	1.070700
H	1.869800	1.629290	1.944500
H	0.625600	0.762500	-3.025920
H	-0.851400	1.320400	-2.633990
H	0.149900	4.498500	0.696500
H	0.929300	3.918800	-0.692700

LDL6:

O	0.000000	0.000000	0.000000
O	-2.129000	0.593610	-1.000900
O	0.468700	-2.403470	-0.771800
O	1.836300	0.828930	1.632300
O	-0.921700	-1.086310	2.208900
O	2.007900	-0.144823	-3.242900
H	0.485100	0.728390	0.578800
H	-0.861000	0.291940	-0.266700
H	-2.862100	1.448930	-0.946000
H	-1.947900	0.333800	-1.778900
H	-0.406900	-2.620870	-0.743900
H	0.389500	-1.286470	-0.592100
H	1.499300	1.033530	2.581100
H	2.679200	0.498820	1.816000
H	-0.630400	-0.944190	1.367500
H	-0.687000	-0.297510	2.810700
H	2.208900	0.804840	-3.154300
H	2.886600	-0.313580	-3.029200

LDL7:

O	0.000000	0.000000	0.000000
O	0.696100	1.306290	-1.999900
O	1.091300	-2.340510	0.399650
O	0.581300	1.310790	2.187020
O	-2.606400	0.074210	0.339520
O	3.159597	1.801890	-1.116000
H	0.231500	-0.864910	0.410800
H	0.117400	0.669040	0.638200
H	0.368400	0.907120	-1.254900
H	0.641400	0.916270	-2.909800
H	1.816400	-2.416810	1.221536
H	1.579500	-2.379410	-0.454500
H	1.547500	1.192330	2.115060
H	0.308300	2.291990	2.664600
H	-1.728700	0.084190	0.167900
H	-2.954800	0.684080	-0.215500
H	2.333630	1.460890	-1.451900
H	3.446090	2.642890	-1.489800

LDL8:

O	0.000000	0.000000	0.000000
O	1.211500	0.754760	-2.142700
O	-2.241800	1.329793	0.014600
O	-0.888600	-2.457000	0.296000
O	1.061000	1.265422	2.050000
O	3.126900	-2.474500	-0.218400
H	0.609800	0.356540	0.640200
H	-0.257900	-0.830800	0.170400
H	0.911400	1.645540	-2.455600
H	0.717900	0.282200	-1.295200
H	-2.735800	1.166900	0.923100
H	-1.508000	0.696400	-0.162600
H	-0.539100	-2.626100	1.257300
H	-0.274500	-2.978500	-0.313600
H	0.573400	2.127280	2.174100
H	1.923100	1.331077	2.676600
H	3.342600	-1.688000	-0.793400
H	2.278800	-2.472700	-0.705000

LDL9:

O	0.000000	0.000000	0.000000
O	-2.420100	-0.516500	0.405900
O	0.022000	0.622170	-2.555500
O	1.388400	-2.247900	-0.122100
O	1.037300	2.232260	1.027800
O	2.050130	1.062010	3.200100
H	0.305900	-0.847400	-0.174100
H	-0.863900	-0.230800	0.422500
H	-2.705600	-1.464200	0.332800
H	-2.575500	-0.061300	-0.417700
H	-0.110800	1.556990	-2.499600
H	-0.008900	0.420950	-1.614400
H	1.835890	-3.009300	-0.552100
H	1.963970	-2.136400	0.635700
H	1.813080	2.615300	0.489500
H	0.739100	1.452830	0.491700
H	1.488200	1.489080	2.562600
H	1.462400	0.730970	3.878200

LDL10:

O	0.000000	0.000000	0.000000
O	-0.630400	-2.249300	1.091600
O	-0.827000	0.125060	-2.446000
O	-0.383900	1.823960	1.859000
O	2.330470	0.650010	-1.043300
O	-0.011300	3.820100	-0.275900
H	-0.132300	0.514270	0.882900
H	-0.075300	-0.971500	0.391100
H	-1.486000	-2.934500	1.216100
H	0.182900	-2.734300	1.349300
H	-1.616500	0.560000	-2.951500
H	-0.754200	0.219230	-1.449300
H	0.141600	2.511300	2.384900
H	-1.204400	1.904590	2.396500
H	2.234930	1.015239	-2.012500
H	1.461200	0.456990	-0.656000
H	0.165000	3.158900	0.532400
H	-0.410700	4.499700	0.263500
